# Fermentation of Alginate and Its Oligosaccharides by the Human Gut Microbiota: Structure–Property Relationships and New Findings Focusing on *Bacteroides xylanisolvens*

**DOI:** 10.3390/nu17091424

**Published:** 2025-04-24

**Authors:** Jiayi Li, Youjing Lv, Meng Shao, Depeng Lv, Zhiliang Fu, Peng Guo, Quancai Li, Qingsen Shang

**Affiliations:** 1 Key Laboratory of Marine Drugs of Ministry of Education, Shandong Key Laboratory of Glycoscience and Glycotherapeutics, School of Medicine and Pharmacy, Ocean University of China, Qingdao 266003, China; ljy15634420699@163.com (J.L.); lvyoujing1988@163.com (Y.L.); 2 Laboratory for Marine Drugs and Bioproducts, Qingdao Marine Science and Technology Center, Qingdao 266237, China; 3 Marine Biomedical Research Institute of Qingdao, Qingdao 266071, China; shaomeng@ouc.edu.cn (M.S.); lvdepeng@ouc.edu.cn (D.L.); fuzhiliang@ouc.edu.cn (Z.F.); guopeng1213@ouc.edu.cn (P.G.)

**Keywords:** alginate, alginate oligosaccharides, gut microbiota, fermentation, *B. xylanisolvens*, structure–property relationships

## Abstract

**Background/Objectives:** Alginate and its oligosaccharides (AOS) are widely used in the food industry all over the world. However, how they are fermented by the human gut microbiota has not been fully elucidated. Here, we aim to explore the structure–property relationships of the fermentation of these carbohydrates by the human gut microbiota. **Methods:** High-performance liquid chromatography, 16S rRNA gene amplicon high-throughput sequencing, whole genome sequencing, and metabolome analysis were used to study the fermentation of alginate and AOS by the human gut microbiota. **Results and Conclusions:** Low-molecular-weight alginate and AOS were more fermentable than alginate. Moreover, fermentation of AOS with a molecular weight (Mw) of 0.8 kDa produced higher amounts of acetate and butyrate than that with a Mw of 0.3 kDa. *B. xylanisolvens* was a keystone species responsible for the fermentation. Additionally, each *B. xylanisolvens* strain was characterized with a unique capability for AOS fermentation. Specifically, *B. xylanisolvens* P19-10, a bacterium isolated from healthy human colon, exhibited the best fermentation capacity. Genomic analysis suggested that *B. xylanisolvens* P19-10 was armed with a plethora of carbohydrate-active enzymes. Additionally, the polysaccharide lyase family 6_1 was identified as a candidate enzyme responsible for the utilization of AOS. Moreover, fermentation of AOS by *B. xylanisolvens* P19-10 was associated with significant changes in bacterial metabolites and metabolic pathways. **Future perspectives:** Our study provides novel mechanistic insights into the fermentation of alginate and AOS by human gut microbiota, which has applications for the development of new carbohydrate-based nutraceuticals and foods.

## 1. Introduction

Alginate and its oligosaccharides (AOS) are marine-sourced bioactive carbohydrates with multiple functions [1,2]. Chemically, alginate and AOS are composed of (1, 4) linked β-D-mannuronic acid (M) and α-L-guluronic acid (G). Today, owing to the desired health-promoting effects and physicochemical properties, alginate and AOS are widely used in the food and nutraceuticals industry all over the world [1,2,3].

Previous studies have indicated that the alginate lyase genes were transferred horizontally from the marine phylum Bacteroidota (formerly Bacteroidetes) species to the human gut microbiota in ancient times [4,5]. Therefore, alginate and AOS can be fermented by the human gut microbiota [5,6]. During the past years, accumulating evidence has demonstrated that each enterotype of human gut microbiota is characterized with a unique capability for the fermentation of alginate and AOS [7,8]. In addition, specific microbes from the human gut, such as *Bacteroides xylanisolvens*, *Bacteroides ovatus*, *Bacteroides finegoldii*, *Bacteroides uniformis*, and *Bacteroides thetaiotaomicron* have all been illustrated to be able to utilize and ferment alginate and AOS [9].

However, as microbiota-accessible carbohydrates, although alginate and AOS have been documented to be fermentable by the human gut microbiota [7,8], the structure–property relationships and the detailed fermentation characteristics of alginate and AOS by the human gut microbiota have not been fully characterized. Additionally, the human gut microbiota is a highly complex and dynamic ecosystem that is composed of trillions of different bacteria [10,11,12]. Under such a competitive environment [13,14], which bacterium would stand out as the keystone species for fermentation has also not been elucidated.

In the present study, by using a combination of anaerobic culture and multiomics techniques, we aim to explore the above questions in the research field. We hope that our study will provide novel mechanistic insights into the fermentation of alginate and AOS by the human gut microbiota.

## 2. Materials and Methods

### 2.1. Chemicals and Reagents

The alginate (Alg), low-molecular-weight alginate (LAlg), alginate oligosaccharides 1 (AOS1), and alginate oligosaccharides 2 (AOS2) were all kindly provided by Qingdao Haida Marine Oligose Technology Co., Ltd. (Qingdao, Shandong, China). The molecular weight (Mw) of the alginate was 100 kDa. The Mw of the LAlg was 2.2 kDa. The Mw of the AOS1 was 0.8 kDa. The Mw of the AOS2 was 0.3 kDa.

Hemin and L-cysteine hydrochloride were purchased from Sangon Biotech (Shanghai, China). The standard short-chain fatty acids (SCFAs) solutions (including propionate, succinate, acetate, and butyrate), tryptone, peptone, yeast extract, and Tween 80 were all obtained from Sigma-Aldrich (St. Louis, MO, USA). All other analytical-grade chemicals used in the present study were acquired from Sinopharm Chemical (Shanghai, China).

### 2.2. Fermentation of Alginate and AOS by the Human Gut Microbiota and the Keystone Bacterium B. xylanisolvens

The fresh fecal samples were collected from eight healthy volunteers who resided in Qingdao (Shandong, China). The healthy volunteers included in this study were not athletes. They all had a sedentary lifestyle. Moreover, all the volunteers consumed a low-fat diet in their daily life. Specifically, the volunteers consisted of a 23-year-old female, a 26-year-old female, two 24-year-old females, and four 25-year-old males.

The human experiments for the collection of fecal samples were approved and supported by the Ethical Committee of the Ocean University of China, School of Medicine and Pharmacy (Permission No. OUC-2022-1017-01). The human experiments were conducted according to the International Committee of Medical Journal Editors (ICMJE) guidelines on the Protection of Research Participants.

The well-established VI culture medium was used for the fermentation experiment, as previously described [15]. First, about 10 g of the fecal sample were weighted. After that, the fecal samples were homogenized with sterile phosphate-buffered saline to prepare a 20% (wt/vol) slurry. To start the fermentation, 1 mL of the fecal slurry was inoculated into 50 mL of the VI culture medium. In our study, four different media containing Alg, LAlg, AOS1, and AOS2 as the major carbon source were prepared. Alg, LAlg, AOS1, and AOS2 were added separately to the medium at a final concentration of 8.0 g/L.

All the fermentation experiments were carried out at 37 °C in an Electrotek AW 500SG anaerobic (80% N_2_, 10% H_2_ and 10% CO_2_) chamber (Shipley, West Yorkshire, UK). During the experiment, about 6 mL of the culture medium was collected at different time points (12, 24, 36, and 48 h) to check the utilization of different carbohydrates by the human gut microbiota. The fermentation was terminated after 48 h.

All the *B. xylanisolvens* strains, including *B. xylanisolvens* AY11-1, *B. xylanisolvens* AS29-19, *B. xylanisolvens* P19-10, *B. xylanisolvens* R8-7, *B. xylanisolvens* AY13-1, *B. xylanisolvens* E1-6, *B. xylanisolvens* B17-5, *B. xylanisolvens* B3-32, and *B. xylanisolvens* D1-5 were isolated from the fecal samples of healthy individuals using the enrichment culture method, as previously described [15]. All these strains were available to the authors. Specifically, *B. xylanisolvens* AY11-1 was used as previously described [9]. The VI culture medium containing AOS1 at a final concentration of 8.0 g/L was used for the fermentation experiment.

During the experiment, about 6 mL of the culture medium was collected at different time points (12, 24, 36, 48, 60, and 72 h) to check the utilization of AOS1 by different strains of bacteria. The bacterial growth was monitored by measuring the optical density (OD) of the broth cultures spectrophotometrically at 600 nm using a ReadMax 1200 microplate spectrophotometer from Shanghai Flash Spectrum Biological Technology Co., Ltd. (Shanghai, China). The fermentation was terminated after 72 h.

### 2.3. Carbohydrate Utilization and Fermentation Products Analysis

The phenol–sulfuric acid (PSA) method was applied to investigate the utilization of alginate and AOS during fermentation, as documented previously [15]. The SCFAs concentrations in the culture media were analyzed using an Agilent 1260 high-performance liquid chromatography (HPLC) system (Agilent Technologies, Inc., Santa Clara, CA, USA) equipped with an Aminex HPX-87H column (Bio-Rad, Hercules, CA, USA), as previously described [9,15].

Thin-layer chromatography (TLC) analysis was carried out to investigate the utilization of AOS1 by different *B. xylanisolvens* strains using the protocol described elsewhere [15]. The samples were first loaded onto a pre-coated silica gel-60 aluminum TLC plate (Merck, Darmstadt, Germany) and then resolved using the formic acid/*n*-butanol/water (6:4:1, vol/vol/vol) solution as an eluent. The solution should be used immediately after preparation.

### 2.4. 16S rRNA Gene Amplicon High-Throughput Sequencing and Bioinformatics Analysis

The QIAamp Power Fecal Pro DNA Kit (Qiagen, Hilden, Germany) was used to extract the metagenomic DNA of the human gut microbiota both from the fresh fecal samples and from the fermentation media. The obtained DNA was first checked for quality. After that, the V3 to V4 hypervariable regions of the 16S rRNA gene were specifically amplified using the universal primers 338F (ACTCCTACGGGAGGAGCAG) and 806R (GGACTACHVGGGTWTCTAAT), as previously described [15].

The obtained amplicons of the 16S rRNA gene were sequenced on an Illumina PE300 platform (San Diego, CA, USA) from Majorbio Bio-Pharm Biotechnology (Shanghai, China). Bioinformatic analyses of the sequencing data, including Chao index analysis, Ace index analysis, Shannon index analysis, observed species analysis, principal components analysis (PCA), non-metric multidimensional scaling (NMDS) score plot analysis, heatmap analysis, and Wilcoxon rank-sum test analysis were all conducted using different online tools from the Majorbio Cloud Platform (https://cloud.majorbio.com/).

### 2.5. Whole Genome Sequencing and Bioinformatics Analysis

*B. xylanisolvens* P19-10 was grown in liquid VI culture medium that contained AOS1 as the major carbon source at a final concentration of 8.0 g/L. The genomic DNA of *B. xylanisolvens* P19-10 was extracted using the QIAamp DNA mini kit (Qiagen, Hilden, Germany). After that, the concentration of the obtained DNA was determined using a Thermo Fisher Scientific NanoDrop 2000 spectrophotometer (Thermo Fisher Scientific Inc., Waltham, MA, USA).

The whole genome of *B. xylanisolvens* P19-10 was sequenced on an Illumina HiSeq platform and an Oxford Nanopore Technologies (ONT) Nanopore PromethION platform (Oxford, Cambridge, UK) from the Majorbio Bio-Pharm Biotechnology (Shanghai, China), as previously described [15]. Bioinformatics analyses of the sequencing data, including clusters of orthologous genes (COG) function classification and carbohydrate-active enzyme (CAZymes, http://www.cazy.org/) analysis were all conducted using the online bioinformatic tools from the Majorbio Cloud Platform (https://cloud.majorbio.com/).

### 2.6. Metabolome Analysis

*B. xylanisolvens* P19-10 was cultured in the liquid VI medium that contained AOS1 as the major carbon source at a final concentration of 8.0 g/L. The utilization of AOS1 by *B. xylanisolvens* P19-10 was monitored every 3 h using TLC. The bacteria were collected before (Dpre group) and after (Dpost group) the utilization of AOS1. The metabolites of *B. xylanisolvens* P19-10 were extracted using the cold (4 °C) methanol/acetonitrile (1:1 *v*/*v*) solution.

The untargeted metabolome analysis of *B. xylanisolvens* P19-10 was conducted using the ultra-high-performance liquid chromatography (UHPLC) (Vanquish UHPLC, Thermo) coupled to an LTQ Orbitrap XL mass spectrometer (MS) (Thermo Scientific, Waltham, MA, USA) from Applied Protein Technology (APTBIO) Co., Ltd. (Shanghai, China). The mass spectrum data were acquired under both the negative mode and positive mode.

The metabolites were identified with the help of an in-house database established using available authentic standards from APTBIO. Bioinformatic analyses of the metabolome data including PCA, Volcano plot analysis, and Kyoto Encyclopedia of Genes and Genomes (KEGG) pathway analysis were all performed using the R package ROPLS (Version 1.6.2) and the online tools from the Cloud Platform from Applied Protein Technology (https://bio-cloud.aptbiotech.com/).

### 2.7. Statistical Analyses

All the data in the present study are expressed as the mean ± standard error of mean (SEM). The statistical analyses were performed using a one-way ANOVA (analysis of variance) with post hoc Tukey’s tests (GraphPad Prism Version 8.0.2; San Diego, CA, USA). All results were considered statistically significant at *p* < 0.05 among different groups. * *p* < 0.05; ** *p* < 0.01; *** *p* < 0.001.

## 3. Results

### 3.1. Low-Molecular-Weight Alginate and AOS Are More Fermentable than Alginate by the Human Gut Microbiota

**Figure 1:**
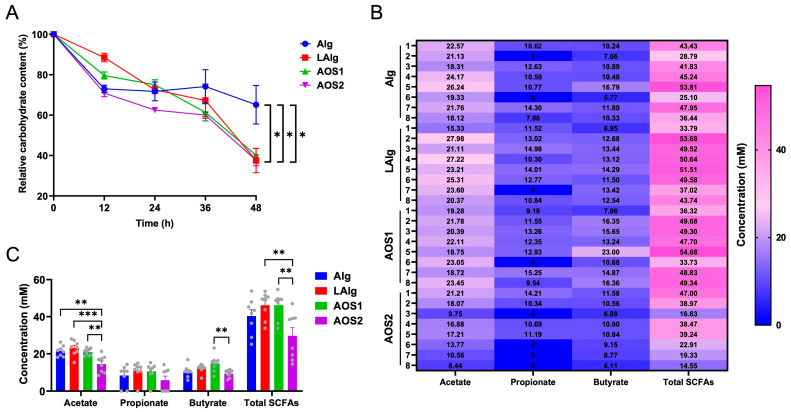
Fermentation of alginate and AOS by the human gut microbiota. Relative carbohydrate content (**A**). Heatmap (**B**) and bar chart (**C**) of the concentrations of different SCFAs in the culture media at 48 h. Alg, fermentation of alginate; LAlg, fermentation of low-molecular-weight alginate; AOS1, fermentation of alginate oligosaccharides 1; AOS2, fermentation of alginate oligosaccharides 2. * *p* < 0.05; ** *p* < 0.01; *** *p* < 0.001.

Alginate and AOS are widely used in the food industry [1,16]. However, how they are fermented by the human gut microbiota has not been fully elucidated. Here, we aimed to explore the effect of Mw on the fermentation outcomes of alginate and AOS. Interestingly, we found that low-molecular-weight alginate and AOS were more fermentable than alginate by the human gut microbiota. For example, the human gut microbiota only fermented about 40% of alginate in the culture medium (Figure 1A). In contrast, about 60% of the low-molecular-weight alginate and AOS were utilized and fermented by the human gut microbiota (Figure 1A).

SCFAs are end fermentation products of dietary polysaccharides and oligosaccharides produced by intestinal microbes [17,18,19]. In the present study, acetate, propionate, and butyrate were identified as predominate SCFAs produced by the human gut microbiota during fermentation of alginate and AOS (Figure 1B,C). Interestingly, further analysis suggested that fermentation of AOS with a molecular weight (Mw) of 0.8 kDa (AOS1) produced higher amounts of acetate and butyrate than that with a Mw of 0.3 kDa (AOS2) (Figure 1B,C). This indicates that the Mw has a very significant effect on the fermentation outcomes of AOS.

### 3.2. B. xylanisolvens Is a Keystone Species for the Fermentation of Alginate and AOS in the Human Gut Microbiota

**Figure 2:**
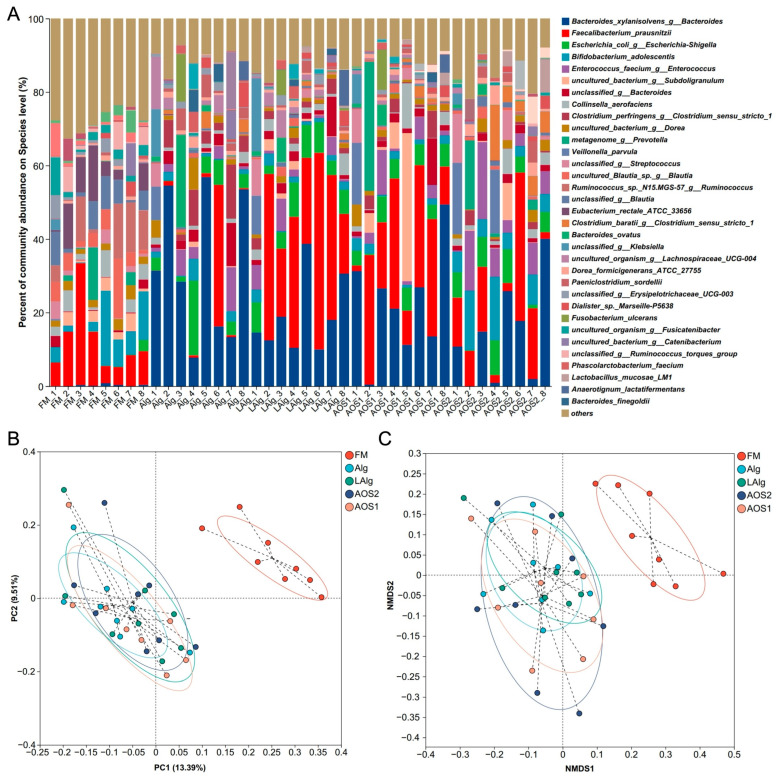
Composition and structure of the human gut microbiota before and after fermentation. Composition of the gut microbiota at the species level (**A**). PCA analysis (**B**) and NMDS analysis (**C**) of the structure of the gut microbiota. FM, fecal microbiota before fermentation; Alg, fermentation of alginate; LAlg, fermentation of low-molecular-weight alginate; AOS1, fermentation of alginate oligosaccharides 1; AOS2, fermentation of alginate oligosaccharides 2.

Given that alginate and AOS can be fermented by the human gut microbiota, we then wondered which was the keystone bacterium responsible for this process. To answer this question, we explored the human gut microbiota using 16S rRNA gene amplicon high-throughput sequencing and bioinformatics analysis. Interestingly and expectedly, we found a dramatic change in the structure of the gut microbiota before and after fermentation (Figure 2A and Figure S1). Some genera were significantly enriched after fermentation, suggesting that these bacteria were candidate keystone species, as they could utilize alginate and AOS as growth substrates.

PCA and NMDS analysis indicated that there was no significant difference in the structure of the gut microbiota after fermentation of alginate and AOS (Figure 2B,C). This indicated that possibly the keystone species for the fermentation of alginate and AOS in the human gut microbiota was shared among different groups.

**Figure 3:**
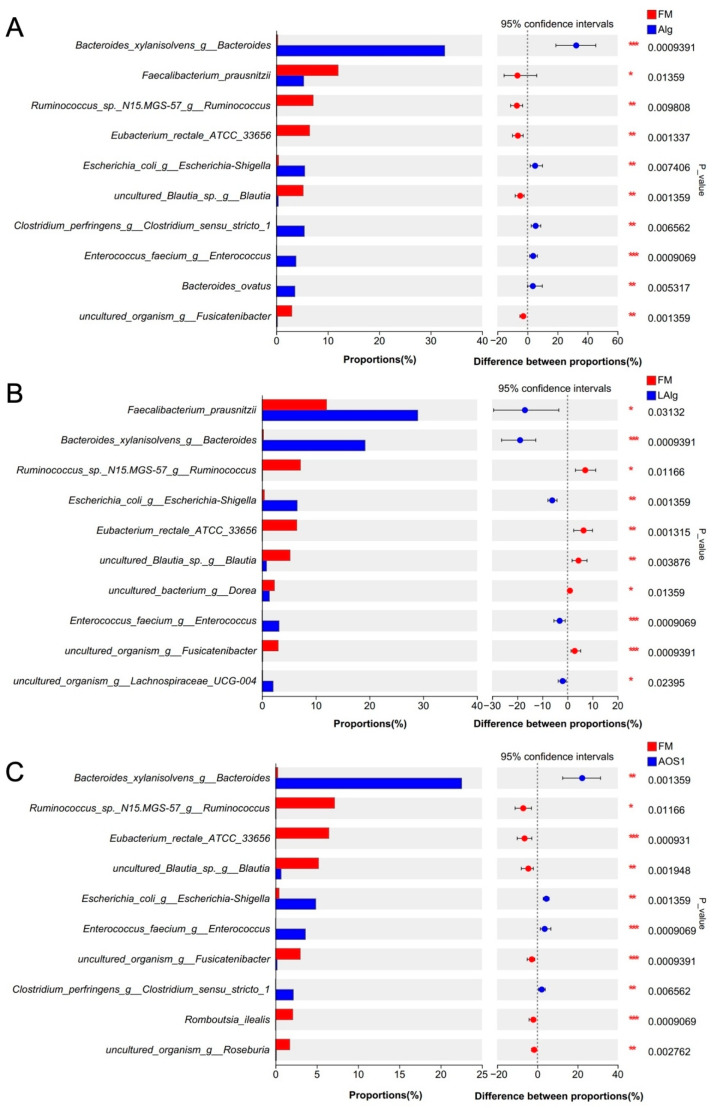
Wilcoxon rank-sum test analysis of the human gut microbiota before and after fermentation. Comparison of the gut microbiota at the species level between FM and Alg (**A**). Comparison of the gut microbiota at the species level between FM and LAlg (**B**). Comparison of the gut microbiota at the species level between FM and AOS1 (**C**).

To further find out the keystone species for the fermentation, we compared the structural differences of the human gut microbiota using Wilcoxon rank-sum test analysis (Figure 3 and Figure S2). In consideration of the above-mentioned findings, we focused on the bacterium whose abundance was consistently increased after fermentation in all four different groups. Intriguingly, we found that the abundance of *B. xylanisolvens* dramatically increased after fermentation of all four different carbohydrates (Figure 3). Specifically, the abundance of *B. xylanisolvens* increased from 0.26% to 32.72% after fermentation of alginate (Figure 3A), to 19.17% after fermentation of low-molecular-weight alginate (Figure 3B), to 22.54% after fermentation of AOS1 (Figure 3C), and to 13.99% after fermentation of AOS2 (Figure S2).

Notably, in most of the cases, we found that *B. xylanisolvens* was the predominant bacterium in the human gut microbiota after fermentation (Figure 3 and S2). This suggested that *B. xylanisolvens* had successfully outcompeted other bacteria for energy and nutrients under alginate- or AOS-rich conditions. In this regard, we proposed that *B. xylanisolvens* was a keystone species for the fermentation of alginate and AOS in the human gut microbiota.

### 3.3. Each B. xylanisolvens Strain Is Characterized with a Unique Capability for AOS Fermentation

Our results suggested that *B. xylanisolvens* is a keystone species for the fermentation of alginate and AOS in the human gut microbiota. Consistent with our findings, previous studies have well demonstrated that the human colon-derived *B. xylanisolvens* could ferment alginate and its derivatives, including polymannuronate and polyguluronate [9]. However, the fermentation characteristics of AOS by human colon-derived *B. xylanisolvens* hasvenot been explored. Therefore, we next sought to investigate how AOS was fermented by the keystone species *B. xylanisolvens*.

Given that AOS1 (Mw = 0.8 kDa) was more fermentable than AOS2 (Mw = 0.3 kDa) (Figure 1B,C), we next chose to use AOS1 as a substrate for fermentation. A total of nine different *B. xylanisolvens* strains, including *B. xylanisolvens* AY11-1, *B. xylanisolvens* AS29-19, *B. xylanisolvens* P19-10, *B. xylanisolvens* R8-7, *B. xylanisolvens* AY13-1, *B. xylanisolvens* E1-6, *B. xylanisolvens* B17-5, *B. xylanisolvens* B3-32, and *B. xylanisolvens* D1-5 were included in the study. All these strains have been previously isolated from the fecal samples of healthy individuals.

**Figure 4:**
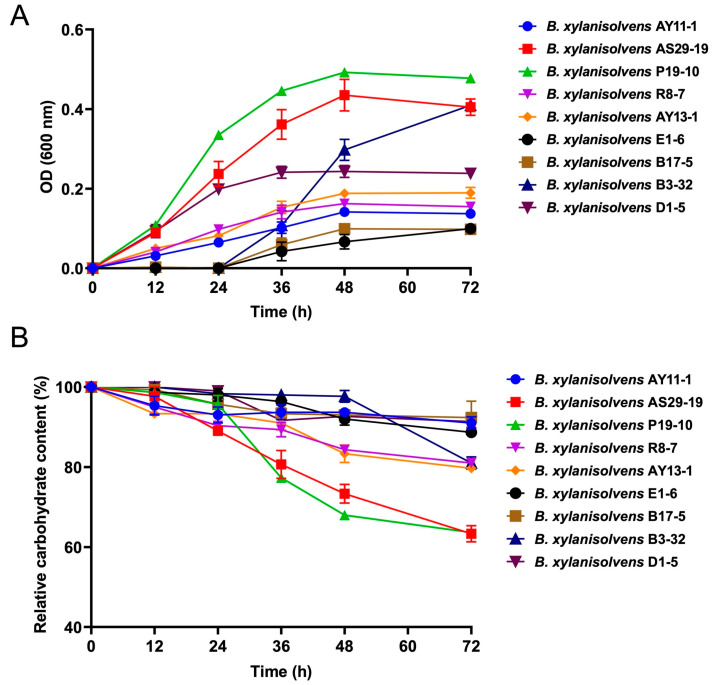
Fermentation of AOS by different strains of *B. xylanisolvens*. Growth curves of the bacterial strains (**A**). Relative carbohydrate content (**B**).

**Figure 5:**
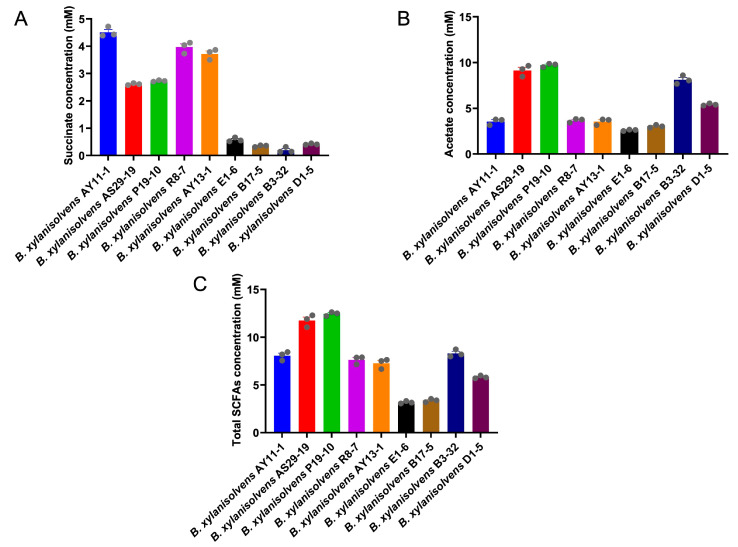
Analysis of the SCFAs in the culture medium of different strains of *B. xylanisolvens*. Succinate concentration (**A**). Acetate concentration (**B**). Total SCFAs concentration (**C**).

Interestingly, we found that each *B. xylanisolvens* strain was characterized with a unique capability for AOS fermentation (Figure 4 and Figure S3). Some strains grew very fast in the liquid culture medium, while others grew very slowly (Figure 4A and Figure S3). Some reached a very high OD value (~0.5), while others only reached a very low OD value (~0.1) (Figure 4A). Similarly, some strains fermented about 40% of the original AOS in the culture medium, while others only fermented about 10% (Figure 4B and Figure S3). Fermentation of AOS by *B. xylanisolvens* produced significant amounts of SCFAs, including succinate and acetate (Figure 5). However, again, this was also strain-specific (Figure 5).

Intriguingly, out of the nine different bacterial strains, *B. xylanisolvens* P19-10 was identified as the most potent AOS-fermenting bacterium (Figure 4 and Figure 5). *B. xylanisolvens* P19-10 reached the highest OD value, consumed the highest amount of AOS, and produced the highest concentration of total SCFAs (Figure 4 and Figure 5). Therefore, we next focused on this specific bacterium to further explore how AOS was fermented and utilized by the human gut *B. xylanisolvens*.

### 3.4. B. xylanisolvens Is Armed with a Plethora of CAZymes, and PL 6_1 Is Identified as a Candidate Enzyme Responsible for the Utilization of AOS

**Figure 6:**
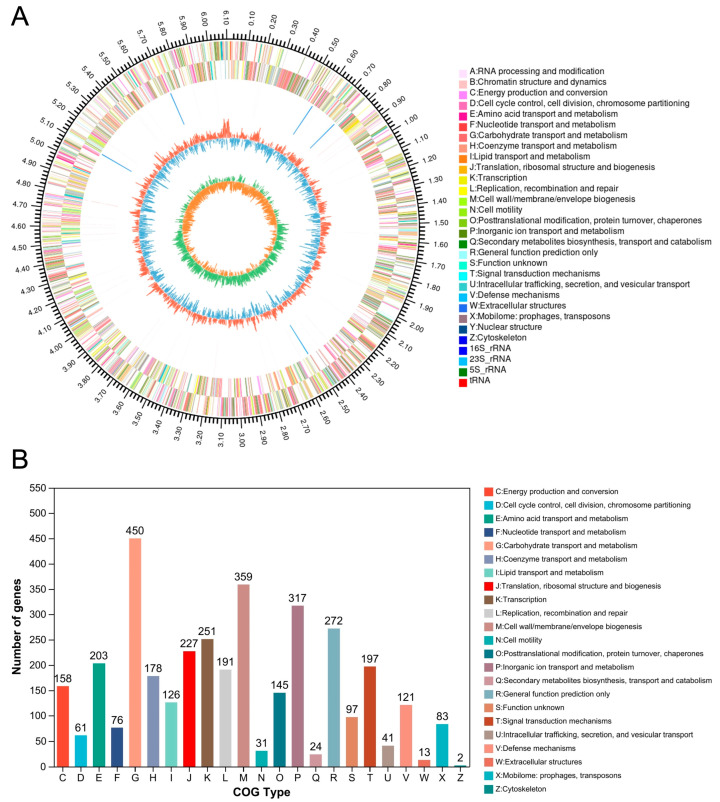
Genomic analysis of *B. xylanisolvens* P19-10. Circos plot showing the genome of *B. xylanisolvens* P19-10 (**A**). COG function classification (**B**).

To uncover how AOS was utilized by *B. xylanisolvens*, we next sequenced the whole genome of the most potent AOS-fermenting bacterium, *B. xylanisolvens* P19-10. The genome size of this specific bacterium was 6,115,302 bp, and the G + C content in the genome was 41.69% (Figure 6A). COG function analysis indicated that the majority of the identified genes in *B. xylanisolvens* P19-10 were involved in the carbohydrate transport and metabolism pathway (Figure 6B), suggesting that this bacterium is very skilled at utilizing dietary carbohydrates in the human gut.

**Figure 7:**
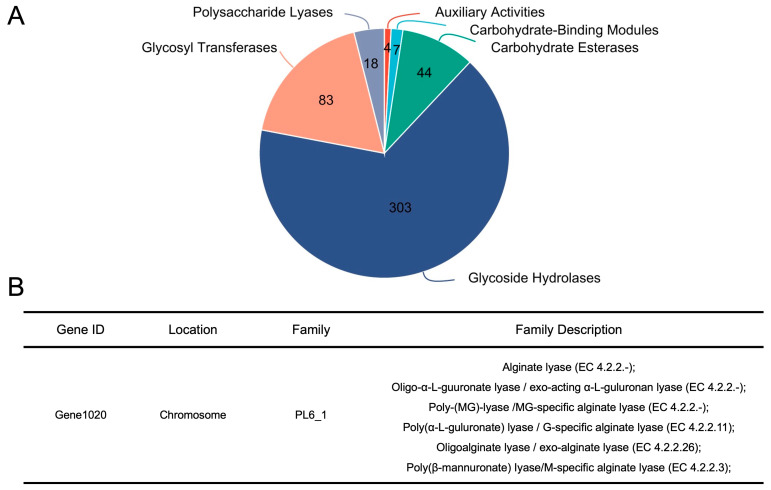
Analysis of the CAZymes in the genome of *B. xylanisolvens* P19-10. Pie chart showing the number of different CAZymes in the genome of *B. xylanisolvens* P19-10 (**A**). Identification of PL 6_1 in the genome of *B. xylanisolvens* P19-10 (**B**).

Previous studies have well demonstrated that bacterial CAZymes can act on dietary carbohydrates and thus initiate the utilization of different glycans [20,21,22]. In this sense, we next investigated the CAZymes in the genome of *B. xylanisolvens* P19-10. Interestingly, a total of 459 different genes were identified as responsible for the expression of CAZymes in the genome of *B. xylanisolvens* P19-10 (Figure 7A). Specifically, these CAZymes included auxiliary activities (AAs), carbohydrate-binding modules (CBMs), carbohydrate esterases (CEs), glycoside hydrolases (GHs), glycosyl transferases (GTs), and polysaccharide lyases (PLs) (Figure 7A).

It is of specific interest to note that among all the different CAZymes, the polysaccharide lyase family 6/subfamily 1 (PL 6_1) was identified in the genome of *B. xylanisolvens* P19-10 (Figure 7B). PL 6_1 is an alginate lyase subfamily, and previous studies have characterized a two-domain PL 6_1 alginate lyase from the human gut microbe *Bacteroides clarus* [23]. In the present study, PL 6_1 was encoded by gene 1020 in the genome of *B. xylanisolvens* P19-10, and, putatively, it had the same alginate lyase activity (Figure 7B). Taken together, our study indicates that PL 6_1 is a candidate enzyme responsible for the utilization of AOS in *B. xylanisolvens* P19-10.

### 3.5. Fermentation of AOS by B. xylanisolvens Is Associated with Significant Changes in Bacterial Metabolites and Metabolic Pathways

**Figure 8:**
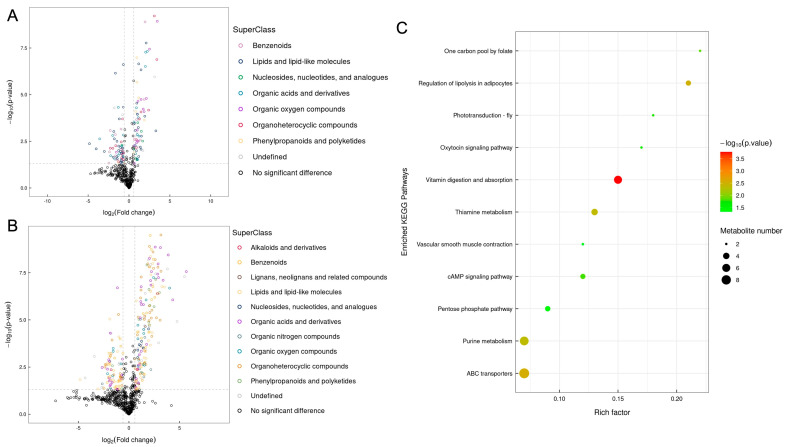
Analysis of the metabolites of *B. xylanisolvens* P19-10 before and after fermentation (Dpost group vs. Dpre group). Volcano plot analysis of the bacterial metabolites data acquired under the negative mode (**A**) and the positive mode (**B**) using MS. Bubble plot showing the enriched KEGG pathways after fermentation (**C**).

Given that AOS could be fermented and utilized by *B. xylanisolvens*, we then wondered what effect it would have on the metabolism of this specific bacterium. To answer this question, we further analyzed the metabolites of *B. xylanisolvens* P19-10 before and after the utilization of AOS. Interestingly, we found that AOS fermentation induced a significant change in the composition of bacterial metabolites (Figures S4–S6). Specifically, organoheterocyclic compounds, organic acids and derivatives, and organic oxygen compounds were upregulated, while lipids and lipid−like molecules were downregulated in response to AOS fermentation (Figure 8A,B).

Consistent with changes in bacterial metabolites, the KEGG analysis indicated that a collection of different metabolic pathways, including vitamin digestion and absorption, thiamine metabolism, purine metabolism, and ABC transporters, were significantly enriched during AOS fermentation by *B. xylanisolvens* P19-10 (Figure 8C). Collectively, these results opened a new window to understand the complex interactions between AOS and the keystone species *B. xylanisolvens*.

## 4. Discussion

### 4.1. Major Findings and Strengths of the Study

Alginate and AOS are widely used in the food industry [2,3,16]. However, how they are fermented by human gut microbiota has not been fully elucidated. In the present study, by using a combination of anaerobic culture and multiomics techniques, we explored in detail the fermentation characteristics of alginate and AOS by the human gut microbiota.

**Figure 9:**
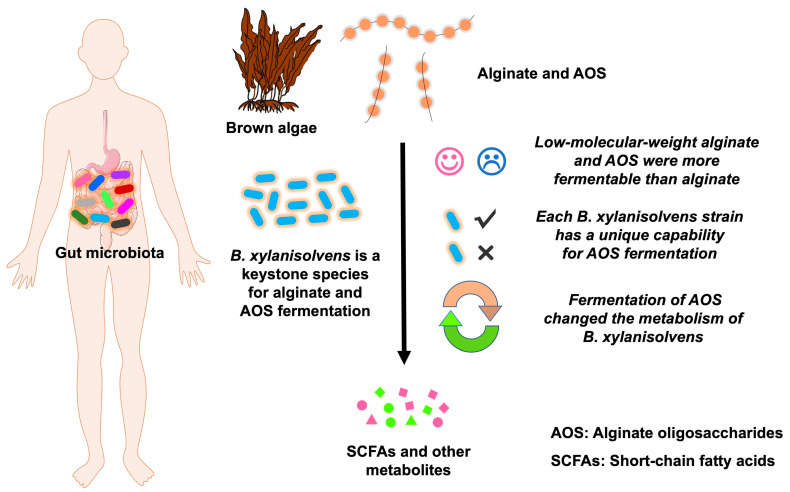
Schematic diagram illustrating the fermentation characteristics of alginate and AOS by the human gut microbiota and the keystone species *B. xylanisolvens*. The symbol “✓” indicates that some *B. xylanisolven* strains are very skilled at fermenting AOS. The symbol “✕” indicates that some *B. xylanisolven* strains are less skilled at fermenting AOS.

Our study demonstrates for the first time that *B. xylanisolvens* is a keystone species for the fermentation of alginate and AOS in the human gut microbiota (Figure 9). Moreover, each *B. xylanisolvens* strain is characterized with a unique capability for AOS fermentation. Our study provides novel mechanistic insights into the fermentation of alginate and AOS by the human gut microbiota, which has applications for the development of new carbohydrate-based nutraceuticals and foods.

### 4.2. Future Directions in This Field

*B. xylanisolvens* has been proposed as a next-generation probiotic bacterium in the human gut microbiota [24,25,26]. Specifically, oral intake of *B. xylanisolvens* has been demonstrated to be effective for the treatment of ulcerative colitis [9], nonalcoholic fatty liver disease [27], and smoking-related nonalcoholic steatohepatitis [28]. Our study suggests that AOS is a good carbon source for the growth of this specific bacteria in the human gut. Future studies are therefore encouraged to explore the beneficial effects of AOS as a next-generation prebiotic ingredient.

The interactions between dietary carbohydrates and the human gut microbiota are very complex [29,30,31,32]. Our study indicates that PL 6_1 is a candidate enzyme responsible for the utilization of AOS in *B. xylanisolvens*. However, further studies are still needed to fully characterize how AOS is fermented by this specific bacterium. Given that the majority of the identified genes in *B. xylanisolvens* are involved in the carbohydrate transport and metabolism pathway, it is anticipated that other proteins might also be involved in capturing and transporting AOS into the bacterial cells. This could be the topic of future research.

Previous studies have demonstrated that the human colon-derived *B. xylanisolvens* is very skilled at fermenting and utilizing alginate and its derivatives [9]. In the present research, we have extended this notion by showing that the capability of *B. xylanisolvens* for the utilization of AOS is strain-specific. Moreover, among different fecal strains of *B. xylanisolvens* tested in vitro, *B. xylanisolvens* P19-10 was identified as the most potent AOS-degrader in our study. More studies are therefore anticipated to further explore the complex interactions between *B. xylanisolvens* P19-10 and different kinds of AOS in vivo.

In our study, for the first time, we demonstrated that AOS fermentation by *B. xylanisolvens* can upregulate the production of organoheterocyclic compounds, organic acids and derivatives, and organic oxygen compounds, and downregulate lipids and lipid-like molecules. In addition, we also observed that specific pathways, including vitamin digestion and absorption, thiamine metabolism, purine metabolism, and ABC transporters were significantly enriched during AOS fermentation by *B. xylanisolvens*. Collectively, these results suggest that AOS supplementation in the diet might be used for targeted modulation of the metabolism of *B. xylanisolvens* in the human colon. However, more detailed studies are needed to verify this hypothesis.

Previous studies have found that fermentation of whole brown seaweeds by the human gut microbiota produced significant amounts of organic acids [33]. Organic acid compounds, especially SCFAs, are key bacterial metabolites produced in the human colon [18,19]. In our study, we observed that fermentation of alginate and AOS by the keystone bacterium *B. xylanisolvens* produced remarkable amounts of acetate and succinate. Acetate and succinate have multiple functions in the distal gut [6,18,19,34], and these metabolites, as produced by the probiotic bacterium *B. xylanisolvens* [9,25], might be able to mediate the beneficial effects of alginate and AOS observed in human clinical trials [35,36]. This is an interesting topic and could be the subject of future research.

### 4.3. Limitations of the Study

Our study has some limitations. First, it is well established that the composition of the human gut microbiota is highly variable across different individuals [37,38]. However, in our study, only eight healthy volunteers were included. The sample size was small, and the results might not be very representative. Therefore, more detailed studies with a larger sample size are needed.

Second, this research is just an in vitro study. In particular, we only used in vitro anaerobic fermentation to explore the utilization of carbohydrates by the human gut microbiota. Human clinical trials and other in vivo studies are needed to confirm the role of *B. xylanisolvens* in the fermentation of alginate and AOS.

Third, although we identified in our study that the organoheterocyclic compounds, organic acids and derivatives, and organic oxygen compounds were upregulated in *B. xylanisolvens* during fermentation of AOS, the molecular mechanisms underlying this phenomenon are largely unknown. More meticulous studies are needed to uncover the biosynthetic pathways of these compounds in *B. xylanisolvens*.

## 5. Conclusions

In conclusion, low-molecular-weight alginate and AOS were more fermentable than alginate. Fermentation of AOS with a Mw of 0.8 kDa produced higher amounts of acetate and butyrate than that with a Mw of 0.3 kDa. *B. xylanisolvens* was a keystone species for the fermentation of alginate and AOS. Moreover, each *B. xylanisolvens* strain was characterized with a unique capability for AOS fermentation. Specifically, *B. xylanisolvens* P19-10, a bacterium isolated from the healthy human colon, exhibited the best fermentation capacity. *B. xylanisolvens* P19-10 was armed with a plethora of carbohydrate-active enzymes. Additionally, the polysaccharide lyase family 6_1 was identified as a candidate enzyme responsible for the utilization of AOS. Moreover, fermentation of AOS by *B. xylanisolvens* P19-10 was associated with significant changes in bacterial metabolites and metabolic pathways.

## Data Availability

The data are available on request from the corresponding authors.

## Supplementary Materials

The following supporting information can be downloaded at: https://www.mdpi.com/article/10.3390/nu17091424/s1, Figure S1. α-diversity analysis of the human gut microbiota before and after fermentation. Chao index (A). Ace index (B). Shannon index (C). Observed species (D). FM, fecal microbiota before fermentation; Alg, fermentation of alginate; LAlg, fermentation of low-molecular-weight alginate; AOS1, fermentation of alginate oligosaccharides 1; AOS2, fermentation of alginate oligosaccharides 2. *** *p* < 0.001; Figure S2. Wilcoxon rank-sum test analysis of the human gut microbiota at the species level before and after AOS2 fermentation; Figure S3. TLC analysis showing the utilization of AOS by different strains of *B. xylanisolvens*; Figure S4. PCA plot analysis of the metabolites of *B. xylanisolvens* P19-10 before and after fermentation (Dpost group vs. Dpre group). The bacterial metabolite data were acquired under the negative mode (A) and the positive mode (B) using MS; Figure S5. Volcano plot analysis of the bacterial metabolite data acquired under the negative mode using MS (Dpost group vs. Dpre group). The upregulated metabolites are marked in red. The downregulated metabolites are marked in blue; Figure S6. Volcano plot analysis of the bacterial metabolite data acquired under the positive mode using MS (Dpost group vs. Dpre group). The upregulated metabolites are marked in red. The downregulated metabolites are marked in blue.
