# Supplementary material for: Fermentation of Alginate and Its Oligosaccharides by the Human Gut Microbiota: Structure–Property Relationships and New Findings Focusing on Bacteroides xylanisolvens
Source: Nutrients. 2025 Apr 24;17(9):1424. doi: 10.3390/nu17091424 (PMC12074081; doi:10.3390/nu17091424)
Supplement: Supplementary file 1 [file nutrients-17-01424-s001.zip › nutrients-3587067-supplementary.pdf]

Fermentation of alginate and its oligosaccharides by the human gut microbiota: structure-property relationships and new findings focusing on *Bacteroides xylanisolvens*

Jiayi Li [1, 2], Youjing Lv [1, 3], Meng Shao [3], Depeng Lv [3], Zhiliang Fu [3], Peng Guo [3], Quancai Li [1, 3 *], Qingsen Shang [1, 2, 3 *]

1 Key Laboratory of Marine Drugs of Ministry of Education, Shandong Key Laboratory of Glycoscience and Glycotechnology, School of Medicine and Pharmacy, Ocean University of China, Qingdao 266003, China;

2 Laboratory for Marine Drugs and Bioproducts, Qingdao Marine Science and Technology Center, Qingdao 266237, China;

3 Marine Biomedical Research Institute of Qingdao, Qingdao, 266071, China;

*Corresponding authors:

Quancai Li, School of Medicine and Pharmacy, Ocean University of China, Qingdao, 266003, China; E-Mail: liquancai@ouc.edu.cn

Qingsen Shang, School of Medicine and Pharmacy, Ocean University of China, Qingdao, 266003, China; E-Mail: shangqingsen@ouc.edu.cn

Supplementary Figures and Tables

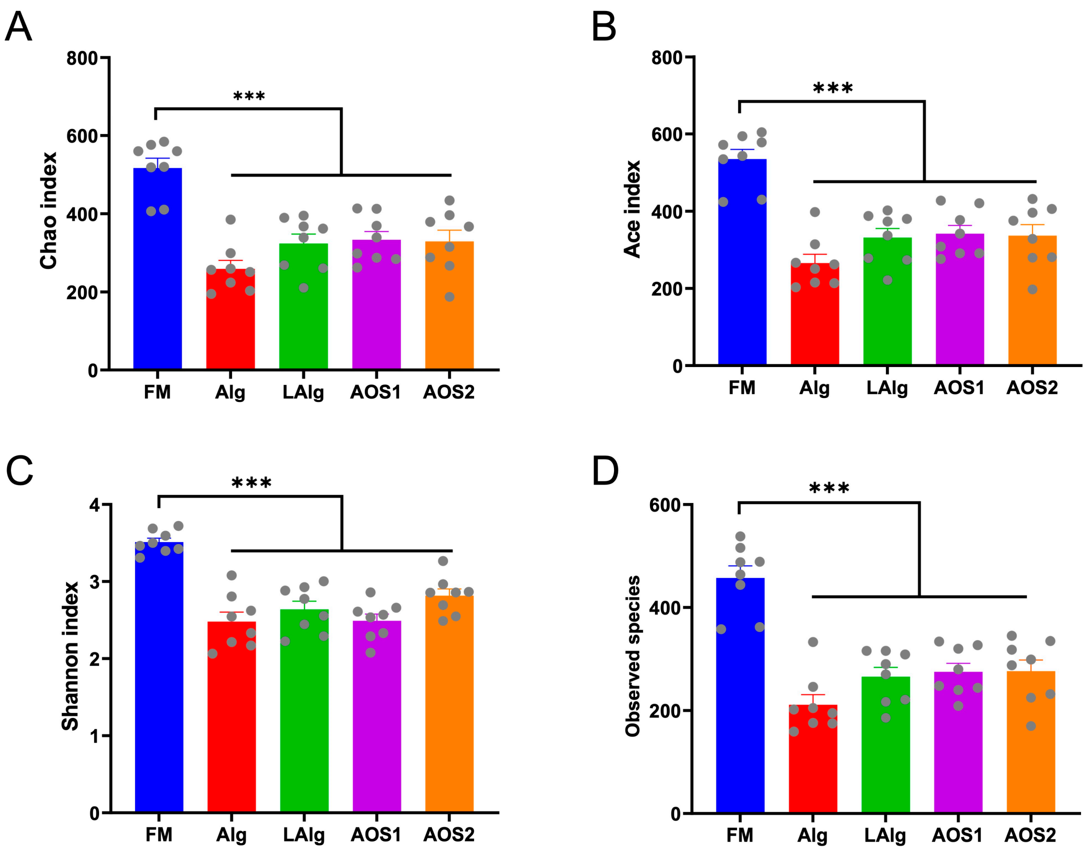

Figure S1. α-diversity analysis of the human gut microbiota before and after fermentation. Chao index (A). Ace index (B). Shannon index (C). Observed species (D). FM, fecal microbiota before fermentation; Alg, fermentation of alginate; LAlg, fermentation of low-molecular-weight alginate; AOS1, fermentation of alginate oligosaccharides 1; AOS2, fermentation of alginate oligosaccharides 2. ***$P < 0.001$.

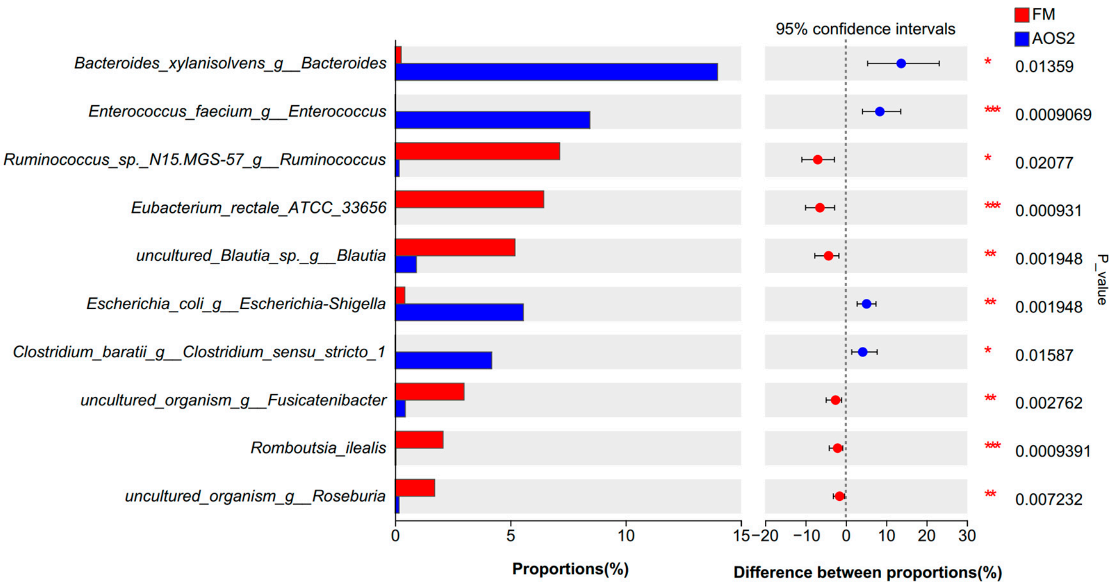

Figure S2. Wilcoxon rank-sum test analysis of the human gut microbiota at the species level before and after AOS2 fermentation.

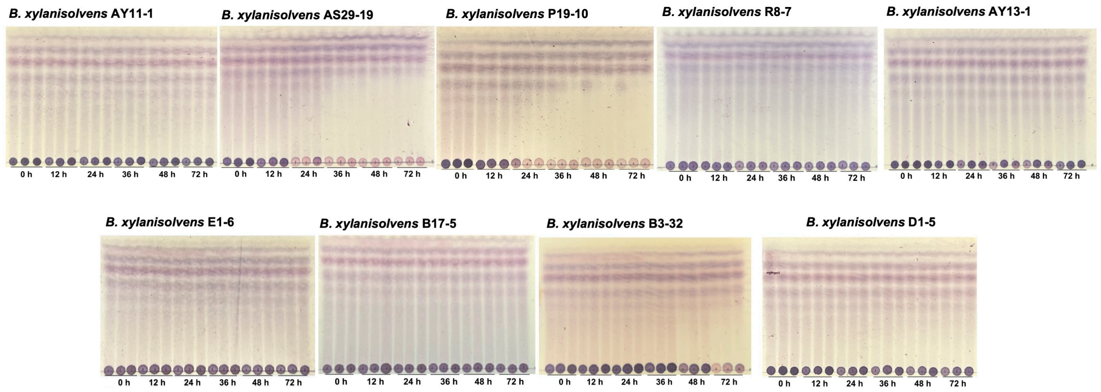

Figure S3. TLC analysis showing the utilization of AOS by different strains of *B. xylanisolvens*.

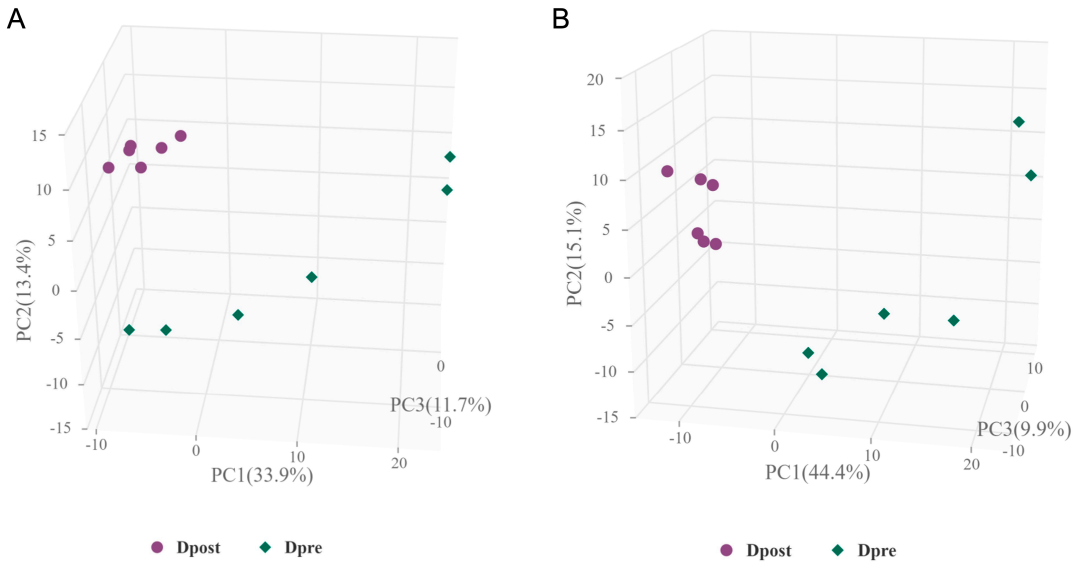

Figure S4. PCA plot analysis of the metabolites of *B. xylanisolvens* P19-10 before and after fermentation (Dpost group vs. Dpre group). The bacterial metabolites data were acquired under the negative mode (A) and the positive mode (B) using MS.

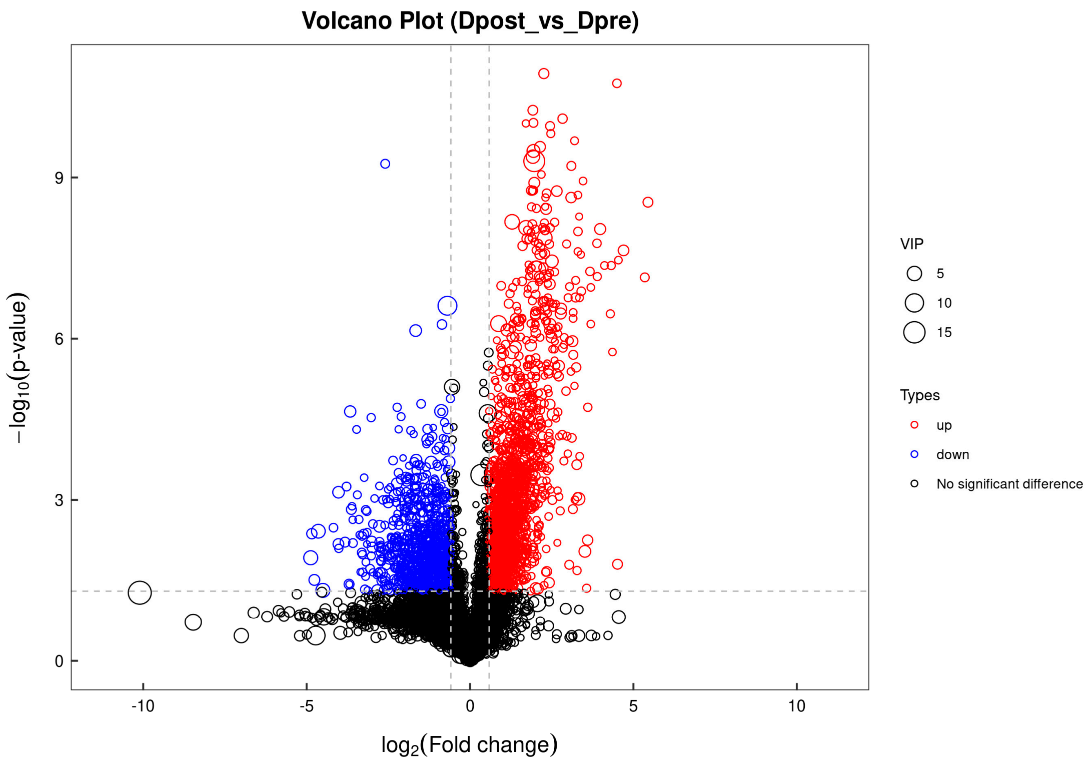

Figure S5. Volcano plot analysis of the bacterial metabolites data acquired under the negative mode using MS (Dpost group vs. Dpre group). The upregulated metabolites were marked in red. The downregulated metabolites were marked in blue.

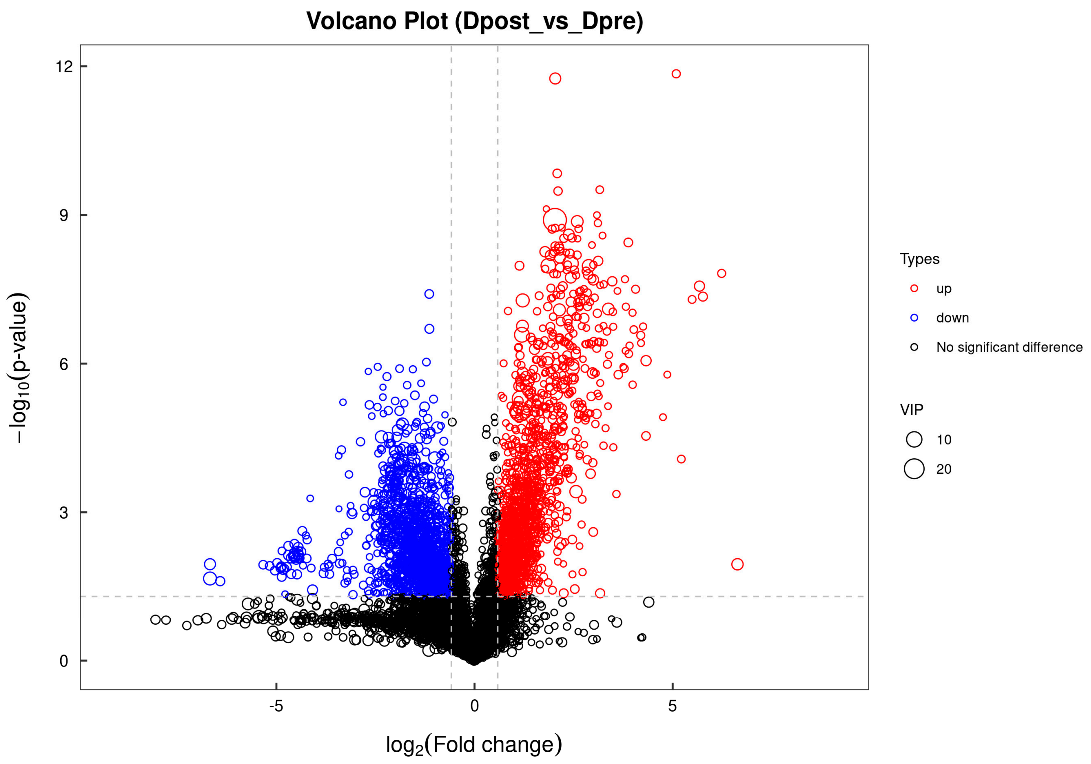

Figure S6. Volcano plot analysis of the bacterial metabolites data acquired under the positive mode using MS (Dpost group vs. Dpre group). The upregulated metabolites were marked in red. The downregulated metabolites were marked in blue.
